# Estimation of own and cross price elasticities of alcohol demand in the UK—A pseudo-panel approach using the Living Costs and Food Survey 2001–2009^☆^

**DOI:** 10.1016/j.jhealeco.2013.12.006

**Published:** 2014-03

**Authors:** Yang Meng, Alan Brennan, Robin Purshouse, Daniel Hill-McManus, Colin Angus, John Holmes, Petra Sylvia Meier

**Affiliations:** a School of Health and Related Research (ScHARR), University of Sheffield, Sheffield, UK; b Department of Automatic Control and Systems Engineering, University of Sheffield, Sheffield, UK

**Keywords:** Alcohol demand, Elasticities, Cross price elasticities, Pseudo-panel

## Abstract

The estimation of price elasticities of alcohol demand is valuable for the appraisal of price-based policy interventions such as minimum unit pricing and taxation. This study applies a pseudo-panel approach to the cross-sectional Living Cost and Food Survey 2001/2–2009 to estimate the own- and cross-price elasticities of off- and on-trade beer, cider, wine, spirits and ready-to-drinks in the UK. A pseudo-panel with 72 subgroups defined by birth year, gender and socioeconomic status is constructed. Estimated own-price elasticities from the base case fixed effect models are all negative and mostly statically significant (*p* < 0.05). Off-trade cider and beer are most elastic (−1.27 and −0.98) and off-trade spirits and on-trade ready-to-drinks are least elastic (−0.08 and −0.19). Estimated cross-price elasticities are smaller in magnitude with a mix of positive and negative signs. The results appear plausible and robust and could be used for appraising the estimated impact of price-based interventions in the UK.

## Highlights

• A pseudo-panel approach is used to estimate own- and cross-price elasticities of off- and on-trade alcoholic beverages.
• Estimated own-price elasticities are all negative, with off-trade cider and beer being most elastic and off-trade spirits and on-trade ready-to-drinks least elastic.
• Estimated cross-price elasticities are smaller in magnitude with a mix of positive and negative signs.
• The results could be used for appraising the estimated impact of price-based interventions such as minimum unit pricing and taxation in the UK.

## 1 Introduction

The consumption of alcohol and the related health and social harms are an issue of extensive policy debate in the UK and many other countries. Price-based policy interventions, such as minimum unit pricing and increases in taxation, have been actively considered by the UK and Scottish governments who aim to reduce harmful alcohol consumption and consequently various alcohol related harms among the population (HM Government, 2012). The estimation of price elasticities of alcohol demand is essential for the appraisal of such price-based policy interventions, because they link the prices of alcohol, which these interventions directly affect, and the demand for alcohol, which such interventions aim to reduce.

It is important to estimate elasticities for different beverage types (e.g., beer vs. wine) and different trade sectors (off-trade e.g., supermarkets vs. on-trade e.g., pubs) for policy appraisals because differential consumer preferences mean elasticities may vary across these categories and because prices and taxes are different for the different beverage types and sectors. Since changes in the price of one beverage type/sector could affect demand for others, it is also important to estimate both own-price and cross-price elasticities. That is, we aim to estimate own-price elasticities to enable us to quantify the percentage change in the demand for one type of alcohol due to a 1% change in the price of this type of alcohol, and cross-price elasticities to quantify the percentage change in demand for one type of alcohol due to a 1% change in the price of another type of alcohol. The cross-price elasticities estimated also allow us to identify whether two types of alcohol of interest are substitutes (i.e., positive sign) or complements (i.e., negative sign).

Previous meta-analyses have focused on differential elasticities by beverage type and demonstrate that beer, wine and spirits have different own-price elasticities, with beer appearing to be less elastic than wine and spirits (Fogarty, 2010; Gallet, 2007; Wagenaar et al., 2009). Cross-price elasticities, especially between off- and on-trade, are less widely studied. Previous studies suggested that different beverage types can be either substitutes or complements (Huang, 2003; Ogwang and Cho, 2009; Ruhm et al., 2012); whilst off-trade purchasing and on-trade-purchasing were typically substitutes, albeit with some exceptions (Collis et al., 2010; Huang, 2003; Purshouse et al., 2010). Few UK studies have investigated cross-price elasticities between off- and on-trade alcohol. Huang et al. examined own- and cross-price elasticities for 4 beverage categories (off-trade beer, on-trade beer, wine and spirits) using aggregate time series data in the UK from 1970 to 2002 (Huang, 2003). Collis et al. used a Tobit approach to model own- and cross-price elasticities for 10 beverage categories (off- and on-trade beer, cider, wine, spirits and ready-to-drinks (RTDs)) using household-level repeated cross-sectional data (the Living Cost and Food Survey, or LCF) in the UK from 2001/2 to 2006 (Collis et al., 2010). When modelling the effects of minimum unit pricing for alcohol, Purshouse et al. used the same cross-sectional data to estimate own- and cross-price elasticities for 16 beverage categories (off- and on-trade beer, wine, spirits and RTDs, further split by high- and low-priced) using an iterative three-stage least squares regression on a system of 17 simultaneous equations (Purshouse et al., 2010). A recent study examined long-run own- and cross-price elasticities specifically for off- and on-trade beer using aggregate time series data from 1982 to 2010 (Tomlinson and Branston, 2014). The key methodological limitation of these studies is the use of either national aggregate time series data which has the problem of small numbers of observations and lack of granularity (thus restricting the number and type of parameters which can be estimated) or cross-sectional data which potentially has severe endogeneity problems. The ideal data source would be longitudinal panel data where individuals or households have repeated observations on both purchases and prices paid over time. Such individual-level panel data would have the advantage that individuals themselves can be used as controls to account for unobserved heterogeneity between individuals and stronger causal inferences can be made. However, individual-level panel data is generally more difficult and costly to obtain than cross-sectional or aggregate time series data. Compared to repeated cross-sectional data, it also suffers more from nonresponse and attrition and normally has smaller sample size and shorter time series.

One solution to the lack of UK individual-level panel data is to use repeat cross-sectional data to construct a pseudo-panel. A pseudo-panel is constructed so that population subgroups rather than individuals become the unit of analysis. Subgroups are defined by a set of characteristics (e.g. birth year, gender, ethnicity) which do not change or remain broadly constant over time. It is assumed that although the individuals within groups change between waves of cross-sectional surveys, the group itself can be viewed as a consistent panel ‘member’ over time. Different ways to define the subgroups of the pseudo-panel can be tested, for example having larger numbers of groups with each having a smaller sample size but greater within-group homogeneity, or smaller numbers of groups with each having a bigger sample size but more within-group heterogeneity. Standard techniques for analysing panel data are then applied (Deaton, 1985; Moffitt, 1993; Verbeek, 2008; Verbeek and Vella, 2005). The pseudo-panel approach has been applied in many empirical studies estimating elasticities of demand for various goods (e.g. Dargay and Vythoulkas, 1999), however, it has not been used to estimate elasticities of alcohol demand.

This study aims to apply the pseudo-panel approach using the LCF data from 2001/2 to 2009 to estimate the own- and cross-price elasticities of 10 categories of beverage (off- and on-trade separated for beer, cider, wine, spirits and RTDs) in the UK. The key research questions are (1) What are the own- and cross-price elasticities for different types of alcohol in the UK? (2) How do the estimates compare with previous estimates from the literature? (3) How robust are these estimates to different model specifications and alternative constructions of the pseudo-panel.

## 2 Methods

### 2.1 Data

The LCF, previously known as the Expenditure and Food Survey, is a national UK survey sponsored by the Office for National Statistics (ONS) and the Department for Environment, Food and Rural Affairs (DEFRA). The LCF is a cross-sectional survey of private households, collecting information on purchasing at both the household and individual level. Data on the purchasing of non-durable goods including alcohol is collected via a confidential two-week personal diary for individuals aged 16 and over. In the UK, around 12,000 households per year are selected and the response rate is typically just over 50%. At the time of the analysis, LCF data was available for the 9 years from 2001 to 2009 (financial years were used for LCF 2001/2 to 2005/6 and this changed to calendar years from 2006) covering 107,763 individuals in 57,646 households in the UK. We obtained the datasets from the UK Data Archive at the University of Essex and detailed data sources are listed in Appendix 1.

Individual-level quantities of alcohol purchased are not available in the standard version of the dataset. However, via a special data request to DEFRA, we obtained anonymised individual-level diary data on both expenditure (in pence) and quantity (in millilitres of product, e.g., 330 ml of beer) for 25 types of alcohol, e.g., off-trade lagers and continental beers (see Appendix 2 for complete list). For this analysis, the 25 types of alcohol were grouped into 10 categories (off- and on-trade separated for beer, cider, wine, spirits and RTDs). The spending during the diary period and the corresponding purchase level (measured in units of alcohol, where 1 unit equals 10 ml of ethanol in the UK) were derived for each of the 10 categories of alcohol for each individual. Alcohol units were calculated by multiplying the recorded volume of product (e.g., 330 ml of beer) and the alcohol by volume (ABV) for each of the 25 beverage types (see Appendix 2 for ABV assumptions). For each individual, mean pence per unit paid (PPU) was calculated for each beverage type by dividing the total spending by the total units purchased. Outliers were defined as individuals who pay extremely high or low PPU for any of the 10 types of alcohol (above 99.5th or below 0.5th percentile of the distributions) and were excluded from the analysis.

### 2.2 Constructing the pseudo-panel

It is important that the subgroups in a pseudo-panel are defined by characteristics that are time-invariant such as the year of birth, gender and ethnicity (Verbeek, 2008). A trade-off also needs to be considered when deciding the number of subgroups (denoted by *C*) in a pseudo-panel: a larger *C* increases the heterogeneity of the pseudo-panel by increasing the variations between subgroups, but also decreases the average number of individuals per subgroup (denoted by *n*_*c*_) resulting in less precise estimates of the subgroup means. Given a fixed total number *N* of individuals in the repeated cross-sectional dataset over time periods *T*, by definition, *N* = *C* × *n*_*c*_ × *T* (for a balanced panel where every panel member has observation for every time period) or *N* = *C* × *n*_*c*_ × T* (for an unbalanced panel where some panel members have missing observations for some time periods), where T^*^ represents the mean number of repeated observations per subgroups. A large *n*_*c*_ is important for the necessary asymptotic theory to be applicable to the pseudo-panel approach (Moffitt, 1993; Verbeek and Vella, 2005) and previous empirical applications of the pseudo-panel approach normally have *n*_*c*_ over 100 (Verbeek, 2008).

In the base case, a pseudo-panel with 72 subgroups was defined by 12 birth cohorts (born between year 1930–1934, and subsequent 5 year intervals, until 1985–1989), gender and 3 socioeconomic groups – higher, middle and lower (see Appendix 3 for definitions). The resulting average number of individuals per subgroup, or *n*_*c*_, is 140 with *N* = 90,652, *C* = 72 and *T* = 9. Table 1 summarises the characteristics of the subgroups. Subgroup observations with less than 30 individuals were excluded from the analysis to ensure robust estimates of subgroup mean statistics. For example, for the panel member of lower-income males who were born between 1940 and 1944, 5 out of 9 observations were excluded (see Table 1).

Three alternative ways to construct subgroups were tested in sensitivity analysis: 96 subgroups defined by birth cohorts, gender and 4 socioeconomic groups (a separate student/other category), 48 subgroups defined by birth cohorts, gender and 2 regions in the UK (England and rest of UK), and 96 subgroups defined by birth cohorts, gender and 4 regions in the UK (Southern England including London, Scotland, Northern Ireland and rest of UK).

### 2.3 Adjustment to prices, income and consumption

The monthly retail price index (RPI) in the UK was used to derive real term prices of alcohol and income, with December 2009 chosen as the base period (Office for National Statistics, 2012). The income variable used in this study is the household gross weekly income which has been consistently collected in the LCF from 2001/2 to 2009. Alcohol consumption or purchasing estimated from self-reported survey data generally suffers from underreporting (Stockwell et al., 2004). Compared to the UK sales clearance data, the coverage of the LCF ranges from 55% to 66% over the period 2001 to 2009 (HM Revenue and Customs, 2012). We estimated beverage specific coverage rates for each year and applied these factors to adjust the alcohol purchase quantities for each individual in the LCF (see Appendix 4 for the adjustment factors and details of how they were applied).

### 2.4 Dependent and independent variables

For each observation of each subgroup (e.g., higher income male born 1960–1964 in the year 2009), the mean units purchased of the 10 types of alcohol, denoted by *C*_*ijt*_, was used as the dependent variable, where *i* and *j* represent the subgroup and the type of alcohol respectively, and *t* represents the time period.

The main independent variables are the mean PPUs for the 10 beverage types which are specific to each subgroup and time period, denoted by *P*_*ijt*_, and subgroup's mean income, denoted *Income*_*it*_. Four other time-variant independent variables were also tested, namely the proportion of individuals having children, being married, being unemployed, and smoking, denoted by *KID*_*it*_, *MRD*_*it*_, *UNE*_*it*_, and *SMK*_*it*_ respectively. Year dummies were included to control for the annual trend and any potentially omitted independent variables that change linearly over time (e.g., mean age of the subgroup). The square of the mean age of subgroup was also tested to account for a potentially non-linear relationship between alcohol purchase and age.

### 2.5 Model specification and testing

Three commonly used models for analysing panel data were tested: fixed effects models (FEMs), random effects models (REMs) and standard ordinary least squares (OLS) models which are illustrated in Eqs. (1)–(3) respectively (Wooldridge, 2009).

(1) $$y_{it}=x_{it}\beta +a_{i}+\varepsilon_{it}\;(\text{FEMs})$$

(2) $$y_{it}=x_{it}\beta +x_{i}\gamma +a_{i}+\varepsilon_{it}\;(\text{REMs})$$

(3) $$y_{j}=x_{j}\beta +\varepsilon_{j}\;(\text{OLS})$$

where *y*_*it*_ is the dependent variables, *i* and *t* represent individual *i* and the time of observation *t*, *x*_*it*_ is a vector of time-variant variables, *x*_*i*_ is a vector of time-invariant variables, *β* to *γ* are parameter vectors, *a*_*i*_ is the unobserved individual effect specific to individual *i*, and *ɛ*_*it*_ is the error term. For OLS, *y*_*j*_ and *x*_*j*_ are the dependent and a vector of independent variables where *j* is a composite of *i* and *t* (e.g., a panel data set with *i* = 100 and *t* = 5 will result a *j* = 500), and *ɛ*_*j*_ is the error term.

REMs assume no correlation between unobserved individual effect and independent variables, i.e., *Corr*(*x*_*it*_,*a*_*i*_) = 0 ; *Corr*(*x*_*i*_, *a*_*i*_) = 0, and FEMs allow for arbitrary correlation between the individual effect and independent variables. In this study, the individual effect refers to the specific effect for each defined subgroup in the pseudo-panel. It has been argued that FEMs are the natural choice for pseudo-panel data when subgroup averages are based on a large number of individuals (Verbeek and Vella, 2005). The Hausman test was used to test whether the underlying correlation structure favoured the assumption of either FEMs or REMs. OLS models do not account for the longitudinal nature of the data and were tested only for comparative purposes. Models were fitted separately for each type of alcohol.

In this study it was assumed that the adjustment of alcohol demand following changes to prices does not take longer than one year to occur. Therefore, the models estimated are static and do not include lagged dependent variables. It was also assumed that habit persistence and any long-term changes in alcohol-related preferences would be captured by the year and birth cohort dummies (for REMs and OLS models). Birth cohort dummies were not included in FEMs which cannot take time-invariant variables. The standard log–log functional form for the dependent variable and independent variables of PPU and income was applied. Other independent variables were tested as levels (i.e., in its original measurement and not logged). *t*-tests and *F*-tests were used to test the inclusion/exclusion of non-PPU/income independent variables. As an illustration, the unrestricted FEM for off-trade beer (i.e., *j* = off-trade beer) is presented in Eq. (4). Regarding the models for the other 9 types of alcohol, the independent variables are identical to those in Eq. (4), with the dependent variable changing to the type of alcohol of interest (e.g., *InC*(*officider*)_*it*_).

(4) $$\ln\;C{\left(offbeer\right)}_{it}=\beta_{1}\ln\;P{\left(offbeer\right)}_{it}+\beta_{2}\ln P{\left(offcider\right)}_{it}+\beta_{3}\ln P{\left(offwine\right)}_{it}+\beta_{4}\ln P{\left(offspirit\right)}_{it}+\beta_{5}\ln P{\left(offRTD\right)}_{it}+\beta_{6}\ln P{\left(onbeer\right)}_{it}+\beta_{7}\ln P{\left(oncider\right)}_{it}+\beta_{8}\ln P{\left(onwine\right)}_{it}+\beta_{9}\ln P{\left(onspirit\right)}_{it}+\beta_{10}\ln P{\left(onRTD\right)}_{it},\beta_{11}LnIncome_{it}+\beta_{12}KID_{it}{+}_{13}MRD_{it}+\beta_{14}UNE_{it}+\beta_{15}SMK_{it}+\beta_{16}Age_{it}^{2}+\gamma YearDummies+a_{i}+\varepsilon_{it}$$

where, *a*_*i*_ is the unobserved fixed effects specific to subgroup *i*, *β*_1_–*β*_10_ represent the own- and cross-price elasticities for the beverage type of interest (e.g., in Eq. (4), *β*_1_ represents own-price elasticity for off-trade beer, while *β*_2_–*β*_10_ represent cross-price elasticities for off-trade beer).

All models were fitted using the STATA/SE 12.1 software (StataCorp, College Station, TX). To account for the different size of the subgroups, weighted FEMs and OLS models were applied using the mean number of individuals within a subgroup, or *n*_*c*_, as weights.

## 3 Results

### 3.1 Model selection

Hausman tests indicate that correlation exists between the independent variables and unobserved individual effects for off-trade beer and wine, and all five on-trade beverages at the 0.05 significance level (see Table 2). On this basis, we reject the null hypothesis and conclude that the FEMs are more appropriate for modelling this data. The choice of FEMs also agrees with previous literature (Verbeek and Vella, 2005). Table 2 summarises the estimated coefficients, standard errors, and statistical test for the FEMs for the 10 alcohol categories and Appendix 5-1 to 5-10 present and compare the results for FEMs, REMs and OLS models.

*F*-Tests suggested that non-PPU/income independent variables are jointly significant for the majority of FEMs tested. The final chosen base case models were FEMs controlling for prices, income, year dummies, age squared, and the proportions of individuals having children, married, unemployed and smoking.

Correlation among the 10 price independent variables was a concern and, if present, may bias the model estimates. The correlation matrix was calculated and it shows only weak to moderate correlations (ranging from −0.11 to 0.43, with an average of 0.13).The comparison of results from FEMs, REMs and OLS models suggest that different model specifications give broadly similar estimates, both in terms of the positive/negative signs and their statistical significance. For example, the estimated own-price elasticities for off-trade beer range from −0.980 to −1.105 for the three model specifications with all estimates statistically significant.

### 3.2 Estimated own- and cross-price elasticities

Estimated own- and cross-price elasticities for the 10 types of alcohol are presented in Table 3 using the base case models.

The estimated own-price elasticities are all negative and 8 out of 10 are statistically significant; off-trade spirits and on-trade RTDs being the exception. The estimates range from −0.08 (off-trade spirits) to −1.27 (off-trade cider). In the off-trade a wide range of elasticities was seen with beer being most elastic (−0.98) after cider, followed by RTDs (−0.59), wine (−0.38) and spirits (−0.08). In the on-trade, elasticities are generally more similar across beverage types, with spirits being most elastic (−0.89), followed by wine (−0.87), beer (−0.79), cider (−0.59) and RTDs (−0.19). For wine and spirits, the estimated own-price elasticities in the off-trade are smaller than in the on-trade. The opposite is observed for beer, cider and RTDs.

The estimated cross-price elasticities were a mix of positive and negative signs (46 and 44 respectively) and only 6 out of 90 were statistically significant, among which 5 out of 6 have positive signs. *F*-Tests showed cross-price effects are jointly significant for the demand for on-trade wine and spirits, using a significance level of 0.05, and for on-trade beer, using a significance level of 0.1. The magnitude of the estimated cross-price elasticities was much smaller than that of the own-price elasticities. If we only focus on central estimates, most of the estimated cross-price elasticities of on-trade demand with respect to off-trade prices are positive (15 out of 25 in the top right corner of Table 3), which appears to indicate some level of overall substitution effect, i.e., if prices fall in the supermarkets people appear to spend more in the pubs and bars.

Using the base case FEMs, three alternative methods for creating subgroups were tested. Appendix 6 compares the estimated own-price elasticities using these methods and shows that these are broadly similar. For example, the own-price elasticity for off-trade beer was estimated to be −0.98 in the base case, −1.03 for the 96 subgroups defined by 4 social groups, −1.12 for the 48 subgroups defined by 2 regions, and −1.11 for the 96 subgroups defined by 4 regions. This suggests that the estimated elasticities are reasonably robust to different subgroup definitions.

## 4 Discussion

This is the first study to utilise a pseudo-panel approach to estimate price elasticities of demand for alcohol. The final base case FEMs enables estimation of own- and cross-price elasticities for 10 different beverage categories. This granularity is essential for detailed analysis of pricing policies which can affect the various beverage categories differentially. The estimated elasticities are not directly comparable with most previous estimates because the data used is from recent (2001 to 2009) UK population surveys, and because the beverage categories included are more detailed than most previous studies which tend not to separate cider and RTDs, or consider off- vs. on-trade differences. Nevertheless, the estimated own-price elasticities are broadly in line with earlier estimates. Three recent meta-analyses estimated that the simple means of reported elasticities are −0.45 to −0.83 for beer, −0.65 to −1.11 for wine and −0.73 to −1.09 for spirits (Fogarty, 2010; Gallet, 2007; Wagenaar et al., 2009), while standard deviations and ranges of individual estimates for the 3 beverage types are 0.46 (−3 to 1.28), 0.51 (−3 to 0.82) and 0.37 (−4.65 to 0.37) for beer, wine and spirits respectively (Fogarty, 2010) which demonstrated significant variations in estimates. The simple average of beer, wine and spirits own-price elasticities estimated from this study (e.g., average of off- and on-trade beer for beer estimate) are −0.88, −0.63 and −0.49 which are all within one standard deviation (as reported by Fogarty (2010)) of any of the three mean estimates from meta-analyses. In the on-trade, a similar pattern is observed in this study as in previous meta-analyses, in that beer appears to be less elastic than wine or spirits. However, this pattern is not observed in the off-trade, where it was found that beer is more elastic than wine and spirits. Overall, the estimated own-price elasticities are broadly in line with historical estimates, and most modelled beverage types are found to have significant negative elasticities suggesting the pseudo-panel approach is a valid technique for deriving alcohol elasticities.

It is more challenging to compare the estimated cross-price elasticities with previous estimates, especially when the beverages are separated by off- and on-trade, because there are few existing studies for comparison. Out of our 90 estimated cross-price elasticities, only 6 are statistically significant, which might be attributable to chance effects. However, the estimation of cross-price elasticities is still useful because: (1) the estimation of own-price elasticities is improved by controlling for cross-price effects, and (2) they can be jointly statistically significant as has been found in our study for on-trade wine and spirits. The estimated cross-price elasticities appear plausible regarding the expected signs and magnitude, and they enable quantified estimates of cross-price effects when appraising policy interventions.

There are several advantages to the pseudo-panel approach. Previous analyses applying cross-sectional models on cross-sectional data (for example Collis et al., 2010) are likely to have substantial problems with endogeneity. Those time-invariant variables that are omitted from the model and are correlated with alcohol prices will be uncontrolled for in such studies. For example, quality preferences are likely to vary considerably between individuals, affecting both price and quantity purchased, and cross-sectional methods would wrongly attribute these variations to prices. The FEM used in our base case substantially reduces endogeneity problems because all time-invariant independent variables, observed or not, are controlled for on the defined subgroup level. Another potential problem of using cross-sectional data relates to the observation interval. It has been observed that the length of the observation interval (e.g., one week vs. one quarter) may have a significant impact on the magnitude of the resulting elasticity estimates, even for genuine panel data methods (Hill-McManus et al., 2013), and it has been suggested that this could be due to inventory behaviour (Fogarty, 2010). The LCF data has an observation interval of two weeks; however the pseudo-panel approach solves this issue through the use of subgroup average purchase quantities, rather than individual purchase quantities, thus smoothing out the short term (i.e., the two week diary period) irregular purchases that constitute inventory behaviour. The use of subgroup average purchase quantities also avoids the problem of excess zero alcohol purchasing observations in cross-sectional data (Collis et al., 2010; Purshouse et al., 2010).

Nevertheless, panel models cannot remove all endogeneity problems. Price variables could be endogenous due to simultaneity because not only is the purchase level dependent on the price, but also the price could potentially be dependent on the purchase level. It has been found that a heavy drinker who spends a bigger proportion of their income on alcohol tends to choose ‘lower quality’ beverages with low PPU (e.g., cheaper brand, larger container); while a lighter drinker with similar income tends to choose a ‘higher quality’ beverage with a higher PPU, perhaps for better taste or a more-convenient container size (Gruenewald et al., 2006). The LCF data does not provide brand or packaging data, therefore the panel models have not controlled for the brand and packaging preferences which may change over time. The split of off- and on-trade beverages and separation of cider and RTDs in this analysis may alleviate the problem to some extent, but the issue remains a concern. In this study, we used self-reported prices which is the price paid by individuals. In theory, elasticities are defined as the change in demand due to a change in price where the price implicitly means price faced, rather than price paid. As far as we know, no survey has attempted to collect primary data on price faced. However, given current data and evidence, we are clear that the pseudo-panel approach is a substantial advance over, and a better alternative to, cross-sectional methods.

When constructing subgroups in pseudo-panels, we assumed that the socioeconomic status (in the base case) and the region people live (in sensitivity analysis) do not generally change over time. While the validity of these assumptions may be questioned, we think they reasonably hold given the limited time period of the data (2001–2009) and the large size of the regions (2 or 4 regions in UK). Furthermore, the similarity of the results and conclusions obtained from the base case and sensitivity analyses is reassuring. Models tested in this study are static without the inclusion of lagged dependent variables. It has been suggested that the inclusion of lagged dependent variables may compromise the explanatory power of other independent variables (Achen, 2012) and that a significant lagged effect of the dependent variable may be due to omitted variables or measurement error bias rather than a true lagged effect (McKinnish, 2002).

The key implication of this study for decision makers is that they can utilise these elasticities to examine the effects of price-based interventions on alcohol purchasing and alcohol related harms in the UK. The estimated elasticities allow detailed estimation of beverage-specific demand changes due to beverage specific price changes. This is appealing for appraising interventions which have differential price impact on different beverage types such as minimum unit pricing which, by setting a floor to the retail price, will mostly affect cheap alcohol. The majority of the cheap alcohol sold in the UK is off-trade beer, cider, wine and sprites. The estimated own-price elasticities indicate substantial decrease in demand for these beverage types if their prices are increased, e.g., through minimum unit pricing and/or target excise duty increases. Given the strong associations between alcohol consumption and a range of alcohol-related harms, the decrease in demand is likely to translate into reduced mortality, morbidity and wider social harms such as crimes, absence from work and harms to family members.

The pseudo-panel method could also be used to explore elasticities for population subgroups. We have performed exploratory analyses to estimate separate elasticities for population subgroups with regard to purchase level (moderate vs. non-moderate purchasers) and socioeconomic status (lower vs. middle/higher). We split the overall LCF dataset into individuals who are moderate purchasers (i.e., ≤3 or 2 units per day for males/females, according to the current UK drinking guidelines) and non-moderate purchasers. Then FEMs are applied to the two datasets separately. The estimated elasticities are presented in Appendix 7-1 and 7-2. For the socioeconomic analysis, we split the LCF dataset into individuals with lower socioeconomic status and those with middle or higher socioeconomic status. Then FEMs with 24 panel members (2 genders, 12 birth cohorts and no socioeconomic breakdown) and with 48 panel members (2 genders, 12 birth cohorts, and middle and higher socioeconomic groups) were used for the low and higher socioeconomic groups respectively. The estimated elasticities are presented in Appendix 7-3 and 7-4. These subgroup analyses are exploratory in nature as the sample size for panel members is smaller (in the case of moderate vs. non-moderate purchaser analysis) and because the heterogeneity among panel members is reduced due to the smaller panel size (in the case of low vs. higher socioeconomic analysis). Therefore, caution is required when interpreting and applying these elasticities.

The pseudo-panel approach is generalisable and could easily be applied to different countries or settings where large sample repeated cross-sectional data is available. The estimated elasticities are UK-specific and some caution should be exercised if considering applying them to a different context. The method could also be extended to a wider set of products which affect public health, for example tobacco or foods high in fat, salt and sugar.

Future research to link prices faced and prices paid would be valuable if datasets could be obtained or constructed. Large scale and long-term individual-level longitudinal data would be hugely beneficial for better estimates of price elasticities. If possible, such data could also include information regarding the branding and packaging information so that the issues around potential price endogeneity could be addressed in more detail.

In conclusion, this study has developed and implemented a pseudo-panel approach to estimate price elasticities of alcohol demand using repeated cross-sectional data. This approach enables longitudinal aspects of the data available to be taken into account, where previous detailed beverage specific estimates of price elasticities have tended to come from cross-sectional analyses with their associated caveats. The resulting estimates of own- and cross-price elasticities appear plausible and robust and could be used for appraising the estimated impact of price-based interventions in the UK.

## Conflict of interest

None.

## Disclaimer

The Living Cost and Food Survey and The Expenditure and Food Survey are Crown Copyright. Neither the Office for National Statistics, Social Survey Division, nor the Data Archive, University of Essex bears any responsibility for the analysis or interpretation of the data described in this paper.

## Figures and Tables

**Table 1:** Characteristics of the 72 subgroups in the pseudo-panel in the base case.

Birth year	Gender	Higher socioeconomic group		Middle socioeconomic group		Lower socioeconomic group	
		Number of repeated observations^a^	Mean number of individuals per year^b^ (Min, Max)	Number of repeated observations^a^	Number of individuals per year^b^ (Min, Max)	Number of repeated observations^a^	Number of individuals per year^b^ (Min, Max)
1930–1934	Male	8 (0)	3.0 (1, 5)	9 (9)	254.6 (227, 295)	9 (0)	8.7 (5, 14)
1935–1939	Male	9 (0)	9.4 (6, 21)	9 (9)	279.0 (255, 306)	9 (3)	23.8 (9, 47)
1940–1944	Male	9 (4)	26.1 (7, 42)	9 (9)	273.9 (199, 322)	9 (8)	59.0 (27, 104)
1945–1949	Male	9 (9)	55.7 (39, 68)	9 (9)	309.2 (242, 337)	9 (9)	89.3 (75, 108)
1950–1954	Male	9 (9)	69.4 (54, 88)	9 (9)	279.0 (260, 308)	9 (9)	82.9 (69, 100)
1955–1959	Male	9 (9)	88.3 (74, 109)	9 (9)	279.2 (250, 313)	9 (9)	84.3 (73, 95)
1960–1964	Male	9 (9)	91.0 (70, 105)	9 (9)	328.4 (291, 353)	9 (9)	98.2 (75, 132)
1965–1969	Male	9 (9)	96.3 (86, 111)	9 (9)	311.8 (288, 365)	9 (9)	97.9 (76, 113)
1970–1974	Male	9 (9)	86.1 (63, 107)	9 (9)	270.6 (243, 292)	9 (9)	89.8 (73, 104)
1975–1979	Male	9 (9)	58.7 (42, 91)	9 (9)	225.6 (196, 260)	9 (9)	75.3 (59, 91)
1980–1984	Male	9 (9)	39.7 (31, 50)	9 (9)	226.1 (203, 275)	9 (9)	85.8 (66, 108)
1985–1989	Male	9 (6)	34.4 (9, 51)	9 (9)	209.1 (72, 281)	9 (8)	86.2 (15, 111)
1930–1934	Female	9 (0)	2.8 (1, 4)	9 (9)	290.9 (217, 354)	9 (0)	9.7 (5, 16)
1935–1939	Female	9 (0)	7.2 (3, 14)	9 (9)	324.1 (274, 373)	9 (1)	17.1 (7, 30)
1940–1944	Female	9 (0)	18.4 (10, 28)	9 (9)	311.1 (265, 391)	9 (7)	44.1 (16, 96)
1945–1949	Female	9 (8)	43.3 (28, 67)	9 (9)	361.9 (291, 424)	9 (9)	91.2 (57, 128)
1950–1954	Female	9 (9)	63.0 (56, 71)	9 (9)	296.1 (265, 330)	9 (9)	96.0 (87, 109)
1955–1959	Female	9 (9)	83.0 (61, 99)	9 (9)	303.9 (291, 333)	9 (9)	95.0 (79, 119)
1960–1964	Female	9 (9)	94.7 (84, 116)	9 (9)	357.1 (301, 408)	9 (9)	113.3 (85, 136)
1965–1969	Female	9 (9)	97.1 (82, 110)	9 (9)	366.1 (326, 419)	9 (9)	114.4 (100, 149)
1970–1974	Female	9 (9)	88.1 (71, 113)	9 (9)	316.1 (295, 339)	9 (9)	109.9 (88, 144)
1975–1979	Female	9 (9)	66.9 (47, 96)	9 (9)	274.2 (241, 306)	9 (9)	88.0 (72, 106)
1980–1984	Female	9 (9)	43.1 (31, 60)	9 (9)	264.6 (229, 284)	9 (9)	103.7 (82, 134)
1985–1989	Female	9 (4)	32.1 (12, 51)	9 (9)	208.3 (58, 325)	9 (8)	90.2 (25, 115)

Summary		9.0 (6.5)	54.1	9.0 (9.0)	288.4	9.0 (7.5)	77.2

a Value in bracket is the number of repeated observations where the number of individuals within a subgroup at a year equals or bigger than 30.

b Values in bracket are the minimum and maximum number of individuals within a subgroup over the 9 years.

**Table 2:** Estimated coefficients, goodness-of-fit, and statistical tests for the fixed effects models of the demand for 10 beverage categories.

	lnC(off-beer)	lnC(off-cider)	lnC(off-wine)	lnC(off-spirits)	lnC(off-RTDs)	lnC(on-beer)	lnC(on-cider)	lnC(on-wine)	lnC(on-spirits)	lnC(on-RTDs)
lnP(off-beer)	−0.980* (0.18)	−0.189 (0.40)	0.096 (0.17)	−0.368 (0.21)	−1.092 (0.57)	−0.016 (0.20)	−0.050 (0.48)	0.253 (0.22)	0.030 (0.27)	0.503 (0.43)
lnP(off-cider)	0.065 (0.09)	−1.268* (0.23)	0.118 (0.07)	−0.122 (0.11)	−0.239 (0.24)	−0.053 (0.06)	0.093 (0.21)	0.067 (0.09)	−0.108 (0.10)	−0.194 (0.18)
lnP(off-wine)	−0.040 (0.18)	0.736* (0.35)	−0.384* (0.16)	0.363 (0.21)	0.039 (0.32)	−0.245 (0.14)	−0.155 (0.36)	0.043 (0.15)	−0.186 (0.22)	0.110 (0.27)
lnP(off-spirits)	0.113 (0.11)	−0.024 (0.30)	0.163 (0.10)	−0.082 (0.17)	−0.042 (0.29)	0.167 (0.10)	0.406 (0.23)	0.005 (0.14)	0.084 (0.15)	0.233 (0.29)
lnP(off-RTDs)	−0.047 (0.05)	−0.159 (0.11)	−0.006 (0.04)	0.079 (0.06)	−0.585* (0.27)	−0.061 (0.04)	0.067 (0.14)	0.068 (0.07)	−0.179* (0.09)	0.093 (0.16)
lnP(on-beer)	0.148 (0.20)	−0.285 (0.43)	0.115 (0.20)	−0.028 (0.23)	0.803 (0.52)	−0.786* (0.28)	0.867 (0.68)	1.042* (0.38)	1.169* (0.36)	−0.117 (0.50)
lnP(on-cider)	−0.100 (0.09)	0.071 (0.15)	0.043 (0.08)	0.021 (0.14)	0.365 (0.21)	0.035 (0.13)	−0.591* (0.23)	0.072 (0.11)	0.237* (0.12)	0.241 (0.20)
lnP(on-wine)	−0.197 (0.12)	0.094 (0.22)	−0.154 (0.14)	−0.031 (0.17)	−0.093 (0.32)	−0.276 (0.18)	−0.031 (0.26)	−0.871* (0.15)	−0.021 (0.16)	−0.363 (0.20)
lnP(on-spirits)	0.019 (0.12)	−0.117 (0.23)	−0.027 (0.10)	−0.280 (0.16)	−0.145 (0.29)	−0.002 (0.11)	−0.284 (0.29)	0.109 (0.15)	−0.890* (0.19)	0.809* (0.33)
lnP(on-RTDs)	0.079 (0.08)	0.005 (0.16)	−0.085 (0.07)	−0.047 (0.09)	0.369 (0.28)	0.121 (0.09)	−0.394 (0.30)	−0.027 (0.10)	−0.071 (0.12)	−0.187 (0.27)
lnIncome	−0.074 (0.24)	−0.133 (0.52)	−0.156 (0.24)	0.795* (0.32)	0.530 (0.63)	0.409 (0.31)	−0.165 (0.54)	0.264 (0.26)	0.592 (0.37)	−0.418 (0.44)
Age × age	−0.001* (0.00)	−0.002* (0.00)	−0.001* (0.00)	0.000 (0.00)	0.000 (0.00)	0.000 (0.00)	−0.001 (0.00)	0.000 (0.00)	−0.001 (0.00)	0.001 (0.00)
% Have kids	−0.565* (0.23)	−0.109 (0.39)	−1.273* (0.37)	−0.475 (0.24)	−0.843 (0.61)	−1.118* (0.19)	−1.699* (0.53)	−1.347* (0.28)	−1.356* (0.28)	−1.526* (0.37)
% Married	0.938* (0.33)	0.863 (0.85)	0.692* (0.34)	0.161 (0.49)	1.498 (1.12)	−0.412 (0.35)	2.021* (0.84)	0.462 (0.54)	−0.819 (0.61)	−0.737 (1.03)
% Unemployed	0.638 (0.79)	−2.114 (1.63)	−0.044 (1.33)	0.414 (1.07)	−0.410 (1.72)	1.455 (1.14)	0.502 (2.19)	1.196 (1.24)	−0.801 (0.94)	−1.662 (1.51)
% Smoker	1.351* (0.45)	1.511 (0.81)	1.149* (0.44)	0.428 (0.66)	1.096 (1.24)	1.066* (0.42)	0.130 (1.00)	0.574 (0.50)	1.111 (0.60)	1.694* (0.83)
*F*-Test1 (*p*-value)^a^	1.06 (0.41)	1.12 (0.36)	1.03 (0.42)	1.16 (0.34)	1.85 (0.08)	1.99 (0.06)	1.10 (0.37)	2.16* (0.04)	2.16* (0.04)	1.46 (0.19)
*F*-Test2 (*p*-value)^b^	6.43* (0.00)	4.61* (0.00)	5.60* (0.00)	2.54* (0.04)	0.69 (0.63)	12.24* (0.00)	4.25* (0.00)	7.11* (0.00)	9.27* (0.00)	14.18* (0.00)
SSE^c^	45.57	171.29	51.46	80.77	248.03	50.26	283.67	68.32	102.06	166.09
Log-likelihood	−96.79	−440.12	−129.72	−253.39	−496.32	−121.86	−562.65	−207.32	−317.30	−404.74
Hausman-test (*p*-value)	37.46* (0.03)	31.09 (0.12)	126.91* (0.00)	24.22 (0.39)	27.11 (0.25)	50.54* (0.00)	43.53* (0.00)	62.73* (0.00)	54.09* (0.00)	40.55* (0.01)

*Remarks*: The values in parentheses are standard errors.

a *F*-Test for cross-price effects.

b *F*-Test for age, % have children, married, unemployed and smoker.

c Residual sum of squares.

* *p*-Value <0.05.

**Table 3:** Estimated own- and cross-price elasticities of off- and–on trade beer, cider, wine, spirits and RTDs in the UK.

	Purchase									
	Off-beer	Off-cider	Off-wine	Off-spirits	Off-RTDs	On-beer	On-cider	On-wine	On-spirits	On-RTDs
Price										
Off-beer	−0.980*	−0.189	0.096	−0.368	−1.092	−0.016	−0.050	0.253	0.030	0.503
Off-cider	0.065	−1.268*	0.118	−0.122	−0.239	−0.053	0.093	0.067	−0.108	−0.194
Off-wine	−0.040	0.736*	−0.384*	0.363	0.039	−0.245	−0.155	0.043	−0.186	0.110
Off-spirits	0.113	−0.024	0.163	−0.082	−0.042	0.167	0.406	0.005	0.084	0.233
Off-RTDs	−0.047	−0.159	−0.006	0.079	−0.585*	−0.061	0.067	0.068	−0.179*	0.093
On-beer	0.148	−0.285	0.115	−0.028	0.803	−0.786*	0.867	1.042*	1.169*	−0.117
On-cider	−0.100	0.071	0.043	0.021	0.365	0.035	−0.591*	0.072	0.237*	0.241
On-wine	−0.197	0.094	−0.154	−0.031	−0.093	−0.276	−0.031	−0.871*	−0.021	−0.363
On-spirits	0.019	−0.117	−0.027	−0.280	−0.145	−0.002	−0.284	0.109	−0.890*	0.809*
On-RTDs	0.079	0.005	−0.085	−0.047	0.369	0.121	−0.394	−0.027	−0.071	−0.187

* *p*-Value <0.05.
