# Supplementary material for: Estimation of own and cross price elasticities of alcohol demand in the UK—A pseudo-panel approach using the Living Costs and Food Survey 2001–2009
Source: J Health Econ. 2014 Mar;34(100):96–103. doi: 10.1016/j.jhealeco.2013.12.006 (PMC3991422; doi:10.1016/j.jhealeco.2013.12.006)
Supplement: Supplementary file 1 [file mmc1.docx]

**Appendices**

*Appendix 1: Data source*

University of Essex. Institute for Social and Economic Research and National Centre for Social Research, Expenditure and Food Survey, 2001/2002 (EFS) [computer file]. Colchester, Essex: UK Data Archive [distributor], 6 September 2007, SN 4697.

EFS 2002/2003, [computer file]. Colchester, Essex: UK Data Archive [distributor], 6 September 2007, SN 5003.

EFS 2003/2004, [computer file]. Colchester, Essex: UK Data Archive [distributor] , 27 May 2008, SN 5210.

EFS 2004/2005, [computer file]. Colchester, Essex: UK Data Archive [distributor] , 27 May 2008, SN 5375.

EFS 2005/2006, [computer file]. Colchester, Essex: UK Data Archive [distributor] , 31 October 2007, SN 5688.

EFS 2006, [computer file]. Colchester, Essex: UK Data Archive [distributor] , 16 July 2009, SN 5986.

EFS 2007, [computer file]. Colchester, Essex: UK Data Archive [distributor] , 16 July 2009, SN 6118.

Living Cost and Food Survey (LCF) 2008, [computer file]. Colchester, Essex: UK Data Archive [distributor] , 2 June 2011, SN 6385.

LCF 2009, [computer file]. Colchester, Essex: UK Data Archive [distributor] , 27 July 2011, SN 6655.

*Appendix 2: Mapping types of alcohol and conversion factors for each type*

| **Maffcode** | **On/off trade** | **Description in LCF** | **Types of alcohol** | **%ABV** |
| --- | --- | --- | --- | --- |
| 38102 | Off-trade | Beers | off-trade beer | 4.0% |
| 38202 | Off-trade | Lagers and continental beers | off-trade beer | 3.9% |
| 38302 | Off-trade | Ciders and perry | off-trade cider | 4.8% |
| 38402 | Off-trade | Champagne, sparkling wines and wine with mixer | off-trade wine | 11.4% |
| 38403 | Off-trade | Table wine | off-trade wine | 12.8% |
| 38501 | Off-trade | Spirits with mixer | off-trade spirits | 7.4% |
| 38601 | Off-trade | Fortified wines | off-trade wine | 14.5% |
| 38701 | Off-trade | Spirits | off-trade spirits | 40.2% |
| 38801 | Off-trade | Liqueurs and cocktails | off-trade spirits | 33.8% |
| 38901 | Off-trade | Alcopops | off-trade RTDs | 4.7% |
| 270101 | On-trade | Spirits | on-trade spirits | 42.3% |
| 270102 | On-trade | Liqueurs | on-trade spirits | 30.3% |
| 270103 | On-trade | Cocktails | on-trade spirits | 13.4% |
| 270104 | On-trade | Spirits or liqueurs with mixer e.g. gin & tonic, Bacardi & coke | on-trade spirits | 7.8% |
| 270201 | On-trade | Wine (not sparkling) including unspecified 'wine' | on-trade wine | 11.3% |
| 270202 | On-trade | Sparkling wines (e.g. Champagne) and wine with mixer (e.g. Bucks Fizz) | on-trade wine | 9.6% |
| 270203 | On-trade | Fortified wine e.g. sherry, port, vermouth | on-trade wine | 17.5% |
| 270204 | On-trade | Cider or perry - half pint or bottle | on-trade cider | 4.8% |
| 270205 | On-trade | Cider or perry - pint or can or size not specified | on-trade cider | 4.8% |
| 270206 | On-trade | Alcoholic soft drinks (alcopops), and ready-mixed bottled drinks | on-trade RTDs | 4.7% |
| 270301 | On-trade | Bitter - half pint or bottle | on-trade beer | 4.3% |
| 270302 | On-trade | Bitter - pint or can or size not specified | on-trade beer | 4.3% |
| 270303 | On-trade | Lager or other beers including unspecified 'beer' - half pint or bottle | on-trade beer | 5.1% |
| 270304 | On-trade | Lager or other beers including unspecified 'beer' - pint or can or size not specified | on-trade beer | 5.1% |
| 270401 | On-trade | Round of drinks, alcohol not otherwise specified | on-trade beer | 4.9% |

*Appendix 3: Definition of socioeconomic groups*

| Description | 3 socioeconomic groups (base case) | 4 socioeconomic groups (sensitivity analysis) |
| --- | --- | --- |
| Employers in large organisations | Higher | Higher |
| Higher managerial | Higher | Higher |
| Higher professional (traditional ) - employees | Higher | Higher |
| Higher professional (new ) - employees | Higher | Higher |
| Higher professional (traditional ) - self employed | Higher | Higher |
| Higher professional (new ) - self-employ | Higher | Higher |
| Lower professional & higher technical (traditional ) - employees | Medium | Medium |
| Lower professional & higher technical (new) - employees | Medium | Medium |
| Lower professional & higher technical (traditional ) - self employed | Medium | Medium |
| Lower professional & higher technical (new ) - self-employ | Medium | Medium |
| Lower managerial | Medium | Medium |
| Higher supervisory | Medium | Medium |
| Intermediate clerical and administrative | Medium | Medium |
| Intermediate sales and service | Medium | Medium |
| Intermediate technical and auxilary | Medium | Medium |
| Intermediate engineering | Medium | Medium |
| Employers (small organisations, non-prof | Medium | Medium |
| Employers (small - agricultural) | Medium | Medium |
| Own account workers (non-professional) | Medium | Medium |
| Own account workers (agriculture) | Medium | Medium |
| Lower supervisory | Medium | Medium |
| Lower technical craft | Medium | Medium |
| Lower technical process operative | Medium | Medium |
| Semi-routine sales | Lower | Lower |
| Semi-routine service | Lower | Lower |
| Semi-routine technical | Lower | Lower |
| Semi-routine operative | Lower | Lower |
| Semi-routine agricultural | Lower | Lower |
| Semi-routine clerical | Lower | Lower |
| Semi-routine childcare | Lower | Lower |
| Routine sales and service | Lower | Lower |
| Routine production | Lower | Lower |
| Routine technical | Lower | Lower |
| Routine operative | Lower | Lower |
| Routine agricultural | Lower | Lower |
| Never worked | Lower | Lower |
| Long-term unemployed | Lower | Lower |
| Full-time students | Medium | Student/other |
| Occupations not stated | Medium | Student/other |
| Not classifiable for other reasons | Medium | Student/other |
| Not recorded | Medium | Student/other |

*Appendix 4: Coverage rates of spirits, beer, wine and cider in the LCF compared with sales data from the HMRC between 2001 to 2009*

|  | All | Spirits | Beer | Wine | Cider |
| --- | --- | --- | --- | --- | --- |
| 2001 | 66.1% | 67.0% | 64.1% | 73.3% | 41.7% |
| 2002 | 60.3% | 51.4% | 59.1% | 72.9% | 41.8% |
| 2003 | 58.7% | 51.0% | 55.5% | 73.4% | 42.5% |
| 2004 | 57.1% | 50.0% | 55.3% | 68.8% | 34.2% |
| 2005 | 57.0% | 46.2% | 54.4% | 71.9% | 35.5% |
| 2006 | 57.1% | 52.4% | 54.2% | 69.7% | 34.1% |
| 2007 | 56.3% | 51.2% | 53.1% | 67.0% | 40.1% |
| 2008 | 55.3% | 51.5% | 51.3% | 67.5% | 35.2% |
| 2009 | 55.5% | 50.0% | 54.3% | 65.9% | 35.4% |

*Example of how coverage rate was estimated*

For year 2001, the annual per capita purchase quantity measured by litres of ethanol for spirits using the EFS2001/2 is 1.3878 litres. For the same year, the HMRC estimated that the per capital consumption of spirits is 2.07 litres. Therefore, the calculated coverage rate for spirits in year 2001 is 67.0% (i.e., 1.3878 divided by 2.07).

*Example of how coverage rate was applied to adjust the LCF purchase quantities*

For year 2001, the purchase quantity of all spirits were adjusted by dividing the unadjusted value by the estimated coverage rate of 67.0%. For example, if the unadjusted purchase quantity for spirits is 5 units per week in year 2001/2, then the adjusted purchase quantity is around 7.46 units per week (i.e., 5 divided by 67.0%).

*Appendix 5-1: Estimated coefficients, goodness-of-fit, and statistical tests for model selection for the demand of off-trade beer*

| Variables | Fixed Effects Model Coeff. (s.e.) | Random Effects Model  Coeff. (s.e.) | OLS  Coeff. (s.e.) |
| --- | --- | --- | --- |
| lnP(off-beer) | -0.980* (0.18) | -1.024* (0.17) | -1.105* (0.17) |
| lnP(off-cider) | 0.065 (0.09) | 0.124 (0.07) | 0.153* (0.07) |
| lnP(off-wine) | -0.040 (0.18) | -0.068 (0.12) | 0.008 (0.18) |
| lnP(off-spirits) | 0.113 (0.11) | 0.089 (0.11) | 0.179 (0.13) |
| lnP(off-RTDs) | -0.047 (0.05) | -0.020 (0.06) | -0.012 (0.04) |
| lnP(on-beer) | 0.148 (0.20) | 0.180 (0.22) | 0.046 (0.23) |
| lnP(on-cider) | -0.100 (0.09) | -0.079 (0.09) | -0.123 (0.11) |
| lnP(on-wine) | -0.197 (0.12) | -0.129 (0.11) | -0.103 (0.13) |
| lnP(on-spirits) | 0.019 (0.12) | 0.027 (0.11) | 0.079 (0.13) |
| lnP(on-RTDs) | 0.079 (0.08) | 0.120 (0.09) | 0.084 (0.07) |
| lnIncome | -0.074 (0.24) | 0.217 (0.19) | 0.210 (0.18) |
| Age x Age | -0.001* (0.00) | -0.001* (0.00) | -0.001* (0.00) |
| % Have children | -0.565* (0.23) | -0.402* (0.17) | -0.383* (0.18) |
| % Married | 0.938* (0.33) | 0.520 (0.29) | 0.339 (0.26) |
| % Unemployed | 0.638 (0.79) | 0.645 (0.69) | 1.594* (0.73) |
| % Smoker | 1.351* (0.45) | 1.544* (0.39) | 1.065* (0.43) |
| Female^1^ |  | -0.228* (0.05) | -0.225* (0.04) |
| Higher socioeconomic^2^ |  | -0.060 (0.12) | -0.076 (0.11) |
| Lower socioeconomic |  | -0.045 (0.08) | -0.059 (0.07) |
| 1930-1934^3^ |  | 2.208* (0.82) | 1.936* (0.77) |
| 1935-1939 |  | 1.683* (0.64) | 1.405* (0.60) |
| 1940-1944 |  | 1.133* (0.47) | 1.001* (0.42) |
| 1945-1949 |  | 0.612 (0.32) | 0.545 (0.28) |
| 1950-1954 |  | 0.225 (0.18) | 0.234 (0.14) |
| 1960-1964 |  | -0.316 (0.17) | -0.252 (0.14) |
| 1965-1969 |  | -0.651* (0.27) | -0.628* (0.24) |
| 1970-1974 |  | -1.215* (0.36) | -1.134* (0.34) |
| 1975-1979 |  | -1.726* (0.47) | -1.634* (0.45) |
| 1980-1984 |  | -2.185* (0.57) | -2.137* (0.54) |
| 1985-1989 |  | -3.069* (0.67) | -3.111* (0.67) |
| F-test1 (p-value) ^4^ | 1.06 (0.41) |  | 1.59 (0.11) |
| F-test2 (p-value) ^5^ | 6.43* (0.00) |  | 4.79* (0.00) |
| SSE^6^ | 45.57 |  | 56.14 |
| Log-likelihood | -96.79 |  | -153.71 |
| REM: Hausman-test (p-value) |  | 37.46* (0.03) |  |

Remarks: *: p-value <=0.05; 1: reference group - male; 2: reference group - middle socioeconomic group; 3: reference group – born between 1960-1965; 4: F-test for cross-price effects; 5: F-test for age, % have children, married, unemployed and smoker; 6: SSE - Residual sum of squares.

*Appendix 5-2: Estimated coefficients, goodness-of-fit, and statistical tests for model selection for the demand of off-trade cider*

| Variables | Fixed Effects Model Coeff. (s.e.) | Random Effects Model  Coeff. (s.e.) | OLS  Coeff. (s.e.) |
| --- | --- | --- | --- |
| lnP(off-beer) | -0.189 (0.40) | -0.367 (0.35) | -0.301 (0.36) |
| lnP(off-cider) | -1.268* (0.23) | -1.275* (0.13) | -1.228* (0.19) |
| lnP(off-wine) | 0.736* (0.35) | 0.646* (0.28) | 0.723* (0.32) |
| lnP(off-spirits) | -0.024 (0.30) | 0.060 (0.24) | 0.065 (0.28) |
| lnP(off-RTDs) | -0.159 (0.11) | -0.186 (0.12) | -0.144 (0.12) |
| lnP(on-beer) | -0.285 (0.43) | -0.349 (0.47) | -0.505 (0.46) |
| lnP(on-cider) | 0.071 (0.15) | 0.125 (0.19) | 0.101 (0.17) |
| lnP(on-wine) | 0.094 (0.22) | 0.028 (0.22) | 0.132 (0.21) |
| lnP(on-spirits) | -0.117 (0.23) | -0.289 (0.23) | -0.134 (0.21) |
| lnP(on-RTDs) | 0.005 (0.16) | -0.003 (0.19) | 0.019 (0.17) |
| lnIncome | -0.133 (0.52) | -0.200 (0.38) | -0.172 (0.43) |
| Age x Age | -0.002* (0.00) | -0.003* (0.00) | -0.003* (0.00) |
| % Have children | -0.109 (0.39) | 0.046 (0.34) | 0.192 (0.35) |
| % Married | 0.863 (0.85) | -0.752 (0.57) | -0.589 (0.62) |
| % Unemployed | -2.114 (1.63) | -2.935* (1.39) | -1.928 (1.49) |
| % Smoker | 1.511 (0.81) | 0.806 (0.80) | 0.827 (0.85) |
| Female^1^ |  | 0.109 (0.09) | 0.113 (0.08) |
| Higher socioeconomic^2^ |  | -0.062 (0.22) | -0.166 (0.23) |
| Lower socioeconomic |  | 0.018 (0.15) | -0.021 (0.15) |
| 1930-1934^3^ |  | 6.126* (1.65) | 6.202* (1.45) |
| 1935-1939 |  | 4.759* (1.29) | 4.704* (1.12) |
| 1940-1944 |  | 3.775* (0.95) | 3.830* (0.85) |
| 1945-1949 |  | 2.289* (0.63) | 2.365* (0.57) |
| 1950-1954 |  | 0.905* (0.34) | 1.017* (0.30) |
| 1960-1964 |  | -1.096* (0.31) | -1.158* (0.29) |
| 1965-1969 |  | -2.280* (0.52) | -2.241* (0.49) |
| 1970-1974 |  | -3.638* (0.72) | -3.621* (0.67) |
| 1975-1979 |  | -4.966* (0.92) | -5.019* (0.87) |
| 1980-1984 |  | -5.886* (1.12) | -5.859* (1.04) |
| 1985-1989 |  | -7.310* (1.31) | -7.333* (1.23) |
| F-test1 (p-value) ^4^ | 1.12 (0.36) |  | 1.06 (0.39) |
| F-test2 (p-value) ^5^ | 4.61* (0.00) |  | 6.68* (0.00) |
| SSE^6^ | 171.29 |  | 196.17 |
| Log-likelihood | -440.12 |  | -473.75 |
| REM: Hausman-test (p-value) |  | 31.09 (0.12) |  |

Remarks: same as Table in Appendix 5-1.

*Appendix 5-3: Estimated coefficients, goodness-of-fit, and statistical tests for model selection for the demand of off-trade wine*

| Variables | Fixed Effects Model Coeff. (s.e.) | Random Effects Model  Coeff. (s.e.) | OLS  Coeff. (s.e.) |
| --- | --- | --- | --- |
| lnP(off-beer) | 0.096 (0.17) | 0.372 (0.20) | 0.109 (0.20) |
| lnP(off-cider) | 0.118 (0.07) | 0.110 (0.08) | 0.067 (0.08) |
| lnP(off-wine) | -0.384* (0.16) | -0.378* (0.14) | -0.229 (0.20) |
| lnP(off-spirits) | 0.163 (0.10) | 0.150 (0.13) | 0.134 (0.13) |
| lnP(off-RTDs) | -0.006 (0.04) | 0.032 (0.07) | 0.014 (0.05) |
| lnP(on-beer) | 0.115 (0.20) | 0.240 (0.26) | 0.316 (0.32) |
| lnP(on-cider) | 0.043 (0.08) | -0.059 (0.11) | -0.031 (0.11) |
| lnP(on-wine) | -0.154 (0.14) | 0.174 (0.12) | 0.065 (0.17) |
| lnP(on-spirits) | -0.027 (0.10) | 0.020 (0.13) | 0.048 (0.13) |
| lnP(on-RTDs) | -0.085 (0.07) | -0.058 (0.11) | -0.086 (0.07) |
| lnIncome | -0.156 (0.24) | 0.128 (0.21) | 0.094 (0.20) |
| Age x Age | -0.001* (0.00) | -0.001* (0.00) | -0.001* (0.00) |
| % Have children | -1.273* (0.37) | -0.686* (0.18) | -0.964* (0.20) |
| % Married | 0.692* (0.34) | 1.095* (0.31) | 1.384* (0.29) |
| % Unemployed | -0.044 (1.33) | -1.600* (0.77) | -1.566 (1.12) |
| % Smoker | 1.149* (0.44) | 1.361* (0.45) | 1.433* (0.47) |
| Female^1^ |  | 0.580* (0.05) | 0.587* (0.04) |
| Higher socioeconomic^2^ |  | 0.234 (0.12) | 0.252* (0.11) |
| Lower socioeconomic |  | -0.601* (0.08) | -0.581* (0.09) |
| 1930-1934^3^ |  | 2.860* (0.93) | 2.538* (0.80) |
| 1935-1939 |  | 2.157* (0.72) | 1.893* (0.61) |
| 1940-1944 |  | 1.756* (0.53) | 1.364* (0.45) |
| 1945-1949 |  | 1.025* (0.36) | 0.816* (0.29) |
| 1950-1954 |  | 0.402* (0.19) | 0.299* (0.15) |
| 1960-1964 |  | -0.426* (0.18) | -0.311* (0.14) |
| 1965-1969 |  | -0.856* (0.30) | -0.733* (0.24) |
| 1970-1974 |  | -1.504* (0.41) | -1.336* (0.34) |
| 1975-1979 |  | -2.125* (0.52) | -1.946* (0.45) |
| 1980-1984 |  | -3.082* (0.63) | -2.762* (0.54) |
| 1985-1989 |  | -4.225* (0.74) | -3.853* (0.64) |
| F-test1 (p-value) ^4^ | 1.03 (0.42) |  | 0.67 (0.73) |
| F-test2 (p-value) ^5^ | 5.60* (0.00) |  | 14.12* (0.00) |
| SSE^6^ | 51.46 |  | 70.78 |
| Log-likelihood | -129.72 |  | -216.87 |
| REM: Hausman-test (p-value) |  | 126.91* (0.00) |  |

Remarks : same as Table in Appendix 5-1.

*Appendix 5-4: Estimated coefficients, goodness-of-fit, and statistical tests for model selection for the demand of off-trade spirits*

| Variables | Fixed Effects Model Coeff. (s.e.) | Random Effects Model  Coeff. (s.e.) | OLS  Coeff. (s.e.) |
| --- | --- | --- | --- |
| lnP(off-beer) | -0.368 (0.21) | -0.231 (0.23) | -0.443* (0.22) |
| lnP(off-cider) | -0.122 (0.11) | -0.239* (0.09) | -0.098 (0.10) |
| lnP(off-wine) | 0.363 (0.21) | 0.339 (0.19) | 0.428* (0.21) |
| lnP(off-spirits) | -0.082 (0.17) | -0.171 (0.15) | -0.070 (0.19) |
| lnP(off-RTDs) | 0.079 (0.06) | 0.098 (0.08) | 0.112 (0.06) |
| lnP(on-beer) | -0.028 (0.23) | -0.356 (0.31) | -0.147 (0.26) |
| lnP(on-cider) | 0.021 (0.14) | 0.006 (0.12) | 0.023 (0.13) |
| lnP(on-wine) | -0.031 (0.17) | 0.137 (0.14) | 0.038 (0.14) |
| lnP(on-spirits) | -0.280 (0.16) | -0.214 (0.15) | -0.243 (0.14) |
| lnP(on-RTDs) | -0.047 (0.09) | -0.082 (0.13) | -0.092 (0.09) |
| lnIncome | 0.795* (0.32) | 0.438 (0.24) | 0.677* (0.26) |
| Age x Age | 0.000 (0.00) | -0.001 (0.00) | 0.000 (0.00) |
| % Have children | -0.475 (0.24) | -0.285 (0.22) | -0.391 (0.22) |
| % Married | 0.161 (0.49) | 0.179 (0.37) | 0.472 (0.33) |
| % Unemployed | 0.414 (1.07) | 0.275 (0.92) | 0.937 (0.99) |
| % Smoker | 0.428 (0.66) | 0.140 (0.52) | 0.451 (0.54) |
| Female^1^ |  | 0.483* (0.06) | 0.505* (0.05) |
| Higher socioeconomic^2^ |  | -0.380* (0.14) | -0.514* (0.14) |
| Lower socioeconomic |  | -0.056 (0.09) | 0.001 (0.10) |
| 1930-1934^3^ |  | 2.323* (1.09) | 1.220 (0.84) |
| 1935-1939 |  | 1.704* (0.85) | 0.889 (0.66) |
| 1940-1944 |  | 1.171 (0.62) | 0.592 (0.49) |
| 1945-1949 |  | 0.817* (0.41) | 0.407 (0.32) |
| 1950-1954 |  | 0.386 (0.23) | 0.219 (0.17) |
| 1960-1964 |  | -0.267 (0.20) | -0.001 (0.17) |
| 1965-1969 |  | -0.521 (0.34) | -0.090 (0.27) |
| 1970-1974 |  | -1.165* (0.47) | -0.560 (0.38) |
| 1975-1979 |  | -1.476* (0.61) | -0.728 (0.49) |
| 1980-1984 |  | -1.952* (0.74) | -1.094 (0.59) |
| 1985-1989 |  | -2.460* (0.87) | -1.314 (0.71) |
| F-test1 (p-value) ^4^ | 1.16 (0.34) |  | 1.93* (0.05) |
| F-test2 (p-value) ^5^ | 2.54* (0.04) |  | 1.56 (0.17) |
| SSE^6^ | 80.77 |  | 90.07 |
| Log-likelihood | -253.39 |  | -282.50 |
| REM: Hausman-test (p-value) |  | 24.22 (0.39) |  |

Remarks : same as Table in Appendix 5-1.

*Appendix 5-5: Estimated coefficients, goodness-of-fit, and statistical tests for model selection for the demand of off-trade RTDs*

| Variables | Fixed Effects Model Coeff. (s.e.) | Random Effects Model  Coeff. (s.e.) | OLS  Coeff. (s.e.) |
| --- | --- | --- | --- |
| lnP(off-beer) | -1.092 (0.57) | -1.145* (0.50) | -1.108* (0.50) |
| lnP(off-cider) | -0.239 (0.24) | -0.243 (0.20) | -0.100 (0.22) |
| lnP(off-wine) | 0.039 (0.32) | -0.256 (0.36) | -0.206 (0.41) |
| lnP(off-spirits) | -0.042 (0.29) | -0.120 (0.30) | 0.001 (0.29) |
| lnP(off-RTDs) | -0.585* (0.27) | -0.429* (0.15) | -0.556* (0.26) |
| lnP(on-beer) | 0.803 (0.52) | 0.824 (0.60) | 0.897 (0.51) |
| lnP(on-cider) | 0.365 (0.21) | 0.200 (0.25) | 0.264 (0.24) |
| lnP(on-wine) | -0.093 (0.32) | 0.037 (0.30) | 0.076 (0.29) |
| lnP(on-spirits) | -0.145 (0.29) | 0.259 (0.31) | -0.083 (0.35) |
| lnP(on-RTDs) | 0.369 (0.28) | 0.264 (0.26) | 0.330 (0.28) |
| lnIncome | 0.530 (0.63) | 0.666 (0.52) | 1.023 (0.56) |
| Age x Age | 0.000 (0.00) | 0.000 (0.00) | 0.000 (0.00) |
| % Have children | -0.843 (0.61) | -0.877 (0.46) | -0.756 (0.45) |
| % Married | 1.498 (1.12) | 0.723 (0.82) | 1.170 (0.83) |
| % Unemployed | -0.410 (1.72) | -2.574 (1.91) | -1.327 (2.06) |
| % Smoker | 1.096 (1.24) | 0.310 (1.09) | 0.855 (1.14) |
| Female^1^ |  | 0.582* (0.14) | 0.803* (0.12) |
| Higher socioeconomic^2^ |  | -0.081 (0.33) | -0.404 (0.34) |
| Lower socioeconomic |  | 0.613* (0.22) | 0.598* (0.21) |
| 1930-1934^3^ |  | 0.200 (2.18) | -0.922 (2.11) |
| 1935-1939 |  | -0.264 (1.72) | -1.142 (1.68) |
| 1940-1944 |  | 0.067 (1.26) | -0.954 (1.22) |
| 1945-1949 |  | -0.317 (0.84) | -0.986 (0.82) |
| 1950-1954 |  | -0.222 (0.49) | -0.370 (0.45) |
| 1960-1964 |  | 0.598 (0.44) | 0.644 (0.43) |
| 1965-1969 |  | 0.081 (0.72) | 0.374 (0.73) |
| 1970-1974 |  | 0.150 (0.98) | 0.571 (0.98) |
| 1975-1979 |  | -0.082 (1.26) | 0.495 (1.25) |
| 1980-1984 |  | 0.266 (1.54) | 1.208 (1.54) |
| 1985-1989 |  | 0.663 (1.79) | 1.483 (1.81) |
| F-test1 (p-value) ^4^ | 1.85 (0.08) |  | 1.30 (0.23) |
| F-test2 (p-value) ^5^ | 0.69 (0.63) |  | 0.94 (0.45) |
| SSE^6^ | 248.03 |  | 293.91 |
| Log-likelihood | -496.32 |  | -533.41 |
| REM: Hausman-test (p-value) |  | 27.11 (0.25) |  |

Remarks : same as Table in Appendix 5-1.

*Appendix 5-6: Estimated coefficients, goodness-of-fit, and statistical tests for model selection for the demand of on-trade beer*

| Variables | Fixed Effects Model Coeff. (s.e.) | Random Effects Model  Coeff. (s.e.) | OLS  Coeff. (s.e.) |
| --- | --- | --- | --- |
| lnP(off-beer) | -0.016 (0.20) | 0.113 (0.18) | -0.070 (0.23) |
| lnP(off-cider) | -0.053 (0.06) | -0.065 (0.07) | -0.161* (0.08) |
| lnP(off-wine) | -0.245 (0.14) | -0.177 (0.13) | -0.214 (0.18) |
| lnP(off-spirits) | 0.167 (0.10) | 0.141 (0.11) | 0.071 (0.13) |
| lnP(off-RTDs) | -0.061 (0.04) | -0.071 (0.06) | -0.041 (0.06) |
| lnP(on-beer) | -0.786* (0.28) | -0.921* (0.23) | -0.533 (0.38) |
| lnP(on-cider) | 0.035 (0.13) | -0.025 (0.10) | 0.104 (0.12) |
| lnP(on-wine) | -0.276 (0.18) | -0.232* (0.11) | -0.311 (0.18) |
| lnP(on-spirits) | -0.002 (0.11) | 0.103 (0.11) | -0.014 (0.16) |
| lnP(on-RTDs) | 0.121 (0.09) | 0.140 (0.10) | 0.013 (0.10) |
| lnIncome | 0.409 (0.31) | 0.598* (0.21) | 0.538* (0.21) |
| Age x Age | 0.000 (0.00) | 0.000 (0.00) | 0.001* (0.00) |
| % Have children | -1.118* (0.19) | -1.160* (0.18) | -1.039* (0.20) |
| % Married | -0.412 (0.35) | 0.366 (0.33) | 2.044* (0.31) |
| % Unemployed | 1.455 (1.14) | 1.229 (0.73) | 0.454 (1.01) |
| % Smoker | 1.066* (0.42) | 1.313* (0.41) | 2.027* (0.55) |
| Female^1^ |  | -1.996* (0.07) | -1.984* (0.05) |
| Higher socioeconomic^2^ |  | -0.225 (0.15) | -0.278* (0.12) |
| Lower socioeconomic |  | -0.128 (0.10) | -0.163 (0.10) |
| 1930-1934^3^ |  | -0.963 (0.87) | -2.973* (0.97) |
| 1935-1939 |  | -0.628 (0.69) | -2.428* (0.76) |
| 1940-1944 |  | -0.647 (0.51) | -1.802* (0.54) |
| 1945-1949 |  | -0.629 (0.36) | -1.351* (0.36) |
| 1950-1954 |  | -0.372 (0.23) | -0.729* (0.19) |
| 1960-1964 |  | 0.314 (0.21) | 0.657* (0.16) |
| 1965-1969 |  | 0.427 (0.31) | 1.122* (0.29) |
| 1970-1974 |  | 0.452 (0.41) | 1.561* (0.41) |
| 1975-1979 |  | 0.455 (0.51) | 2.156* (0.52) |
| 1980-1984 |  | 0.553 (0.63) | 2.742* (0.64) |
| 1985-1989 |  | 0.301 (0.73) | 2.899* (0.76) |
| F-test1 (p-value) ^4^ | 1.99 (0.06) |  | 1.48 (0.15) |
| F-test2 (p-value) ^5^ | 12.24* (0.00) |  | 11.44* (0.00) |
| SSE^6^ | 50.26 |  | 84.79 |
| Log-likelihood | -121.86 |  | -266.19 |
| REM: Hausman-test (p-value) |  | 50.54* (0.00) |  |

Remarks : same as Table in Appendix 5-1.

*Appendix 5-7: Estimated coefficients, goodness-of-fit, and statistical tests for model selection for the demand of on-trade cider*

| Variables | Fixed Effects Model Coeff. (s.e.) | Random Effects Model  Coeff. (s.e.) | OLS  Coeff. (s.e.) |
| --- | --- | --- | --- |
| lnP(off-beer) | -0.050 (0.48) | -0.330 (0.45) | -0.155 (0.52) |
| lnP(off-cider) | 0.093 (0.21) | 0.053 (0.18) | -0.104 (0.22) |
| lnP(off-wine) | -0.155 (0.36) | -0.187 (0.36) | 0.172 (0.40) |
| lnP(off-spirits) | 0.406 (0.23) | 0.411 (0.28) | 0.316 (0.29) |
| lnP(off-RTDs) | 0.067 (0.14) | 0.126 (0.16) | 0.101 (0.15) |
| lnP(on-beer) | 0.867 (0.68) | 1.379* (0.63) | 1.377 (0.82) |
| lnP(on-cider) | -0.591* (0.23) | -0.220 (0.23) | -0.399 (0.27) |
| lnP(on-wine) | -0.031 (0.26) | -0.061 (0.27) | 0.074 (0.33) |
| lnP(on-spirits) | -0.284 (0.29) | -0.023 (0.28) | -0.255 (0.33) |
| lnP(on-RTDs) | -0.394 (0.30) | -0.149 (0.25) | -0.342 (0.28) |
| lnIncome | -0.165 (0.54) | -0.051 (0.48) | -0.006 (0.53) |
| Age x Age | -0.001 (0.00) | -0.001 (0.00) | -0.001 (0.00) |
| % Have children | -1.699* (0.53) | -0.714 (0.43) | -0.582 (0.42) |
| % Married | 2.021* (0.84) | 1.531* (0.76) | 1.891* (0.75) |
| % Unemployed | 0.502 (2.19) | -1.558 (1.80) | -1.650 (1.88) |
| % Smoker | 0.130 (1.00) | 0.381 (1.01) | 0.975 (1.12) |
| Female^1^ |  | -1.080* (0.12) | -1.155* (0.11) |
| Higher socioeconomic^2^ |  | -0.537 (0.29) | -0.609* (0.31) |
| Lower socioeconomic |  | -0.015 (0.20) | -0.088 (0.24) |
| 1930-1934^3^ |  | 0.594 (2.16) | 0.406 (2.31) |
| 1935-1939 |  | 0.207 (1.70) | -0.099 (1.81) |
| 1940-1944 |  | 0.311 (1.25) | 0.066 (1.42) |
| 1945-1949 |  | 0.211 (0.84) | -0.095 (0.86) |
| 1950-1954 |  | 0.122 (0.46) | -0.035 (0.46) |
| 1960-1964 |  | -0.299 (0.42) | -0.138 (0.42) |
| 1965-1969 |  | -0.417 (0.70) | -0.229 (0.72) |
| 1970-1974 |  | -0.432 (0.95) | -0.258 (1.01) |
| 1975-1979 |  | -0.564 (1.22) | -0.214 (1.30) |
| 1980-1984 |  | -0.322 (1.49) | 0.220 (1.57) |
| 1985-1989 |  | -0.172 (1.75) | 0.184 (1.82) |
| F-test1 (p-value) ^4^ | 1.10 (0.37) |  | 0.74 (0.67) |
| F-test2 (p-value) ^5^ | 4.25* (0.00) |  | 2.97* (0.01) |
| SSE^6^ | 283.67 |  | 356.49 |
| Log-likelihood | -562.65 |  | -618.86 |
| REM: Hausman-test (p-value) |  | 43.53* (0.00) |  |

Remarks : same as Table in Appendix 5-1.

*Appendix 5-8: Estimated coefficients, goodness-of-fit, and statistical tests for model selection for the demand of on-trade wine*

| Variables | Fixed Effects Model Coeff. (s.e.) | Random Effects Model  Coeff. (s.e.) | OLS  Coeff. (s.e.) |
| --- | --- | --- | --- |
| lnP(off-beer) | 0.253 (0.22) | 0.298 (0.22) | 0.211 (0.27) |
| lnP(off-cider) | 0.067 (0.09) | 0.094 (0.09) | -0.118 (0.11) |
| lnP(off-wine) | 0.043 (0.15) | 0.221 (0.16) | 0.327 (0.20) |
| lnP(off-spirits) | 0.005 (0.14) | -0.117 (0.14) | -0.114 (0.18) |
| lnP(off-RTDs) | 0.068 (0.07) | 0.092 (0.08) | 0.041 (0.08) |
| lnP(on-beer) | 1.042* (0.38) | 1.281* (0.28) | 1.481* (0.41) |
| lnP(on-cider) | 0.072 (0.11) | 0.142 (0.12) | 0.175 (0.13) |
| lnP(on-wine) | -0.871* (0.15) | -0.963* (0.13) | -0.839* (0.23) |
| lnP(on-spirits) | 0.109 (0.15) | 0.115 (0.14) | 0.129 (0.17) |
| lnP(on-RTDs) | -0.027 (0.10) | -0.068 (0.12) | -0.064 (0.11) |
| lnIncome | 0.264 (0.26) | 0.520* (0.26) | 0.528* (0.26) |
| Age x Age | 0.000 (0.00) | 0.000 (0.00) | 0.001 (0.00) |
| % Have children | -1.347* (0.28) | -1.077* (0.22) | -0.985* (0.25) |
| % Married | 0.462 (0.54) | 0.869* (0.41) | 2.324* (0.38) |
| % Unemployed | 1.196 (1.24) | 0.161 (0.91) | -1.886 (1.09) |
| % Smoker | 0.574 (0.50) | 0.648 (0.50) | 2.086* (0.58) |
| Female^1^ |  | -0.044 (0.08) | -0.029 (0.06) |
| Higher socioeconomic^2^ |  | 0.135 (0.17) | 0.112 (0.15) |
| Lower socioeconomic |  | -0.694* (0.12) | -0.655* (0.10) |
| 1930-1934^3^ |  | -0.891 (1.08) | -1.959 (1.09) |
| 1935-1939 |  | -0.553 (0.84) | -1.523 (0.86) |
| 1940-1944 |  | -0.294 (0.63) | -1.108 (0.63) |
| 1945-1949 |  | -0.226 (0.44) | -0.694 (0.41) |
| 1950-1954 |  | -0.199 (0.27) | -0.418* (0.20) |
| 1960-1964 |  | 0.267 (0.25) | 0.517* (0.20) |
| 1965-1969 |  | 0.416 (0.38) | 0.864* (0.33) |
| 1970-1974 |  | 0.496 (0.50) | 1.248* (0.46) |
| 1975-1979 |  | 0.630 (0.63) | 1.799* (0.61) |
| 1980-1984 |  | 0.395 (0.77) | 2.036* (0.74) |
| 1985-1989 |  | -0.174 (0.90) | 1.881* (0.86) |
| F-test1 (p-value) ^4^ | 2.16* (0.04) |  | 2.81* (0.00) |
| F-test2 (p-value) ^5^ | 7.11* (0.00) |  | 8.66* (0.00) |
| SSE^6^ | 68.32 |  | 119.66 |
| Log-likelihood | -207.32 |  | -360.34 |
| REM: Hausman-test (p-value) |  | 62.73* (0.00) |  |

Remarks : same as Table in Appendix 5-1.

*Appendix 5-9: Estimated coefficients, goodness-of-fit, and statistical tests for model selection for the demand of on-trade spirits*

| Variables | Fixed Effects Model Coeff. (s.e.) | Random Effects Model  Coeff. (s.e.) | OLS  Coeff. (s.e.) |
| --- | --- | --- | --- |
| lnP(off-beer) | 0.030 (0.27) | -0.078 (0.25) | -0.009 (0.27) |
| lnP(off-cider) | -0.108 (0.10) | -0.113 (0.10) | -0.212 (0.12) |
| lnP(off-wine) | -0.186 (0.22) | -0.348 (0.18) | -0.104 (0.24) |
| lnP(off-spirits) | 0.084 (0.15) | 0.093 (0.16) | 0.042 (0.18) |
| lnP(off-RTDs) | -0.179* (0.09) | -0.117 (0.09) | -0.147 (0.10) |
| lnP(on-beer) | 1.169* (0.36) | 1.029* (0.33) | 1.548* (0.42) |
| lnP(on-cider) | 0.237* (0.12) | 0.260 (0.14) | 0.386* (0.14) |
| lnP(on-wine) | -0.021 (0.16) | -0.021 (0.15) | -0.073 (0.20) |
| lnP(on-spirits) | -0.890* (0.19) | -1.036* (0.16) | -0.911* (0.23) |
| lnP(on-RTDs) | -0.071 (0.12) | -0.146 (0.14) | -0.160 (0.14) |
| lnIncome | 0.592 (0.37) | 0.950* (0.29) | 0.899* (0.32) |
| Age x Age | -0.001 (0.00) | 0.000 (0.00) | 0.001 (0.00) |
| % Have children | -1.356* (0.28) | -1.237* (0.26) | -1.370* (0.26) |
| % Married | -0.819 (0.61) | -0.085 (0.46) | 2.010* (0.53) |
| % Unemployed | -0.801 (0.94) | -0.789 (1.04) | -3.049* (1.20) |
| % Smoker | 1.111 (0.60) | 1.383* (0.58) | 2.382* (0.62) |
| Female^1^ |  | -0.434* (0.09) | -0.480* (0.07) |
| Higher socioeconomic^2^ |  | -0.447* (0.20) | -0.627* (0.18) |
| Lower socioeconomic |  | -0.131 (0.14) | -0.044 (0.12) |
| 1930-1934^3^ |  | 0.370 (1.24) | -2.660* (1.26) |
| 1935-1939 |  | 0.698 (0.97) | -1.931* (0.94) |
| 1940-1944 |  | 0.220 (0.72) | -1.639* (0.71) |
| 1945-1949 |  | -0.051 (0.50) | -1.228* (0.47) |
| 1950-1954 |  | -0.181 (0.30) | -0.718* (0.24) |
| 1960-1964 |  | 0.142 (0.28) | 0.761* (0.22) |
| 1965-1969 |  | 0.248 (0.43) | 1.322* (0.38) |
| 1970-1974 |  | 0.247 (0.57) | 1.861* (0.53) |
| 1975-1979 |  | 0.344 (0.72) | 2.687* (0.69) |
| 1980-1984 |  | 0.597 (0.88) | 3.727* (0.85) |
| 1985-1989 |  | 0.637 (1.03) | 4.337* (1.00) |
| F-test1 (p-value) ^4^ | 2.16* (0.04) |  | 3.17* (0.00) |
| F-test2 (p-value) ^5^ | 9.27* (0.00) |  | 9.63* (0.00) |
| SSE^6^ | 102.06 |  | 154.12 |
| Log-likelihood | -317.30 |  | -430.86 |
| REM: Hausman-test (p-value) |  | 54.09* (0.00) |  |

Remarks : same as Table in Appendix 5-1.

*Appendix 5-10: Estimated coefficients, goodness-of-fit, and statistical tests for model selection for the demand of on-trade RTDs*

| Variables | Fixed Effects Model Coeff. (s.e.) | Random Effects Model  Coeff. (s.e.) | OLS  Coeff. (s.e.) |
| --- | --- | --- | --- |
| lnP(off-beer) | 0.503 (0.43) | 0.487 (0.38) | 0.424 (0.41) |
| lnP(off-cider) | -0.194 (0.18) | -0.201 (0.17) | -0.265 (0.19) |
| lnP(off-wine) | 0.110 (0.27) | 0.116 (0.26) | 0.007 (0.33) |
| lnP(off-spirits) | 0.233 (0.29) | -0.050 (0.23) | 0.123 (0.26) |
| lnP(off-RTDs) | 0.093 (0.16) | -0.095 (0.15) | 0.040 (0.18) |
| lnP(on-beer) | -0.117 (0.50) | 0.060 (0.50) | 0.497 (0.54) |
| lnP(on-cider) | 0.241 (0.20) | 0.386 (0.21) | 0.401 (0.21) |
| lnP(on-wine) | -0.363 (0.20) | -0.458* (0.23) | -0.307 (0.21) |
| lnP(on-spirits) | 0.809* (0.33) | 0.778* (0.26) | 0.666* (0.27) |
| lnP(on-RTDs) | -0.187 (0.27) | -0.070 (0.20) | -0.147 (0.26) |
| lnIncome | -0.418 (0.44) | -0.323 (0.43) | -0.346 (0.44) |
| Age x Age | 0.001 (0.00) | 0.001* (0.00) | 0.001 (0.00) |
| % Have children | -1.526* (0.37) | -1.314* (0.35) | -1.282* (0.39) |
| % Married | -0.737 (1.03) | 0.720 (0.67) | 0.754 (0.73) |
| % Unemployed | -1.662 (1.51) | 0.248 (1.48) | -1.690 (1.52) |
| % Smoker | 1.694* (0.83) | 1.909* (0.88) | 2.416* (0.93) |
| Female^1^ |  | -0.115 (0.10) | -0.138 (0.11) |
| Higher socioeconomic^2^ |  | 0.043 (0.26) | 0.001 (0.26) |
| Lower socioeconomic |  | -0.031 (0.18) | -0.004 (0.19) |
| 1930-1934^3^ |  | -6.449* (2.11) | -6.247* (2.22) |
| 1935-1939 |  | -5.445* (1.65) | -5.349* (1.84) |
| 1940-1944 |  | -3.919* (1.20) | -4.009* (1.29) |
| 1945-1949 |  | -2.522* (0.79) | -2.652* (0.85) |
| 1950-1954 |  | -0.974* (0.44) | -1.005* (0.46) |
| 1960-1964 |  | 1.158* (0.39) | 1.077* (0.38) |
| 1965-1969 |  | 2.083* (0.66) | 1.905* (0.67) |
| 1970-1974 |  | 2.867* (0.91) | 2.672* (0.95) |
| 1975-1979 |  | 3.571* (1.17) | 3.360* (1.23) |
| 1980-1984 |  | 5.121* (1.43) | 4.982* (1.51) |
| 1985-1989 |  | 6.041* (1.67) | 5.886* (1.77) |
| F-test1 (p-value) ^4^ | 1.46 (0.19) |  | 1.61 (0.11) |
| F-test2 (p-value) ^5^ | 14.18* (0.00) |  | 4.92* (0.00) |
| SSE^6^ | 166.09 |  | 200.46 |
| Log-likelihood | -404.74 |  | -444.98 |
| REM: Hausman-test (p-value) |  | 40.55* (0.01) |  |

Remarks : same as Table in Appendix 5-1.

*Appendix 6: Estimated own-price elasticities using 4 different methods for creating subgroups*

|  | Base case: 72 subgroups (by birth cohorts, gender and 3 social groups) | 96 subgroups (by birth cohorts, gender and 4 social groups) | 48 subgroups (by birth cohorts, gender and 2 regions) | 96 subgroups (by birth cohorts, gender and 4 regions) | Mean |
| --- | --- | --- | --- | --- | --- |
| Off-beer | -0.980* | -1.032* | -1.124* | -1.114* | -1.062 |
| Off-cider | -1.268* | -1.285* | -1.301* | -1.244* | -1.275 |
| Off-wine | -0.384* | -0.422* | -0.167 | -0.002 | -0.244 |
| Off-spirits | -0.082 | -0.226 | 0.024 | -0.215 | -0.125 |
| Off-RTDs | -0.585* | -0.630* | -0.329 | -0.262 | -0.451 |
| On-beer | -0.786* | -0.976* | -0.802* | -0.693* | -0.814 |
| On-cider | -0.591* | -0.527* | -0.669 | -0.382 | -0.542 |
| On-wine | -0.871* | -0.620* | -0.255 | 0.079 | -0.417 |
| On-spirits | -0.890* | -0.957* | -0.825* | -0.879* | -0.888 |
| On-RTDs | -0.187 | 0.056 | -0.251 | -0.283 | -0.166 |

Remarks *: p-value <=0.05.

*Appendix 7-1*: Estimated own- and cross-price elasticities of off- and –on trade beer, cider, wine, spirits and RTDs in the UK for moderate purchasers

|  | | Purchase | | | | | | | | | |
| --- | --- | --- | --- | --- | --- | --- | --- | --- | --- | --- | --- |
|  |  | Off-beer | Off-cider | Off-wine | Off-spirits | Off-RTDs | On-beer | On-cider | On-wine | On-spirits | On-RTDs |
| Price | Off-beer | -0.439* | -0.353 | 0.324 | -0.133 | -0.611 | -0.153 | -0.493 | 0.290 | -0.452 | 0.165 |
|  | Off-cider | -0.015 | -0.677* | 0.092 | -0.066 | -0.296 | -0.036 | 0.126 | 0.132 | -0.187 | 0.031 |
|  | Off-wine | -0.093 | 0.208 | -0.418* | -0.455 | 0.270 | -0.066 | -0.217 | -0.063 | -0.051 | 0.327 |
|  | Off-spirits | -0.013 | -0.193 | 0.066 | -0.296* | 0.416* | 0.041 | 0.014 | -0.010 | 0.014 | 0.084 |
|  | Off-RTDs | -0.099 | -0.110 | -0.080 | 0.421* | -0.355* | -0.131* | 0.368* | -0.064 | -0.048 | -0.056 |
|  | On-beer | 0.364 | -0.933 | -0.080 | 0.100 | 0.006 | -0.380 | -0.388 | 0.108 | 0.660 | -0.214 |
|  | On-cider | -0.205 | -0.408* | -0.176 | -0.104 | 0.399 | 0.001 | -0.484* | -0.176 | -0.008 | -0.093 |
|  | On-wine | 0.133 | 0.043 | 0.315* | 0.202 | 0.347 | -0.073 | 0.053 | -0.213 | -0.162 | -0.341 |
|  | On-spirits | -0.242 | 0.164 | -0.046 | 0.268 | 0.116 | -0.020 | -0.030 | 0.309* | -0.183 | 0.154 |
|  | On-RTDs | -0.125 | 0.064 | 0.046 | -0.330 | 0.654* | 0.145 | 0.004 | 0.063 | -0.163 | 0.229 |

Remarks *: p-value <0.05

*Appendix 7-2*: Estimated own- and cross-price elasticities of off- and –on trade beer, cider, wine, spirits and RTDs in the UK for non-moderate purchasers

|  | | Purchase | | | | | | | | | |
| --- | --- | --- | --- | --- | --- | --- | --- | --- | --- | --- | --- |
|  |  | Off-beer | Off-cider | Off-wine | Off-spirits | Off-RTDs | On-beer | On-cider | On-wine | On-spirits | On-RTDs |
| Price | Off-beer | -1.094* | -0.120 | -0.141 | -0.085 | -0.409 | -0.058 | 1.638 | 0.164 | 0.818* | 0.313 |
|  | Off-cider | 0.006 | -1.222* | -0.038 | 0.033 | -0.131 | -0.139 | -0.382 | 0.047 | -0.339 | 0.237 |
|  | Off-wine | 0.443* | 0.907 | 0.358 | -0.128 | 1.508* | -0.272 | 0.463 | 0.273 | -0.801 | -0.523 |
|  | Off-spirits | -0.075 | -0.165 | -0.028 | 0.048 | 0.533 | 0.046 | 0.304 | -0.134 | 0.127 | -0.269 |
|  | Off-RTDs | -0.056 | -0.180 | 0.043 | 0.030 | -0.889* | 0.064 | 0.033 | 0.035 | -0.019 | 0.369 |
|  | On-beer | 0.045 | -0.621 | -0.063 | -0.246 | 0.321 | -0.833* | 1.049 | 0.263 | 0.816 | -1.405 |
|  | On-cider | -0.054 | 0.268 | -0.034 | -0.121 | 0.078 | 0.019 | -0.462 | -0.031 | 0.384* | 0.232 |
|  | On-wine | -0.102 | 0.243 | 0.048 | -0.038 | -1.055* | -0.067 | 0.321 | 0.052 | 0.539* | 0.510 |
|  | On-spirits | -0.169 | -0.540 | -0.010 | -0.199 | -0.247 | -0.385* | -0.479 | 0.009 | -1.102* | 0.563* |
|  | On-RTDs | 0.072 | -0.155 | -0.101 | 0.069 | -0.366 | -0.047 | -0.395 | 0.049 | -0.128 | -0.800* |

Remarks *: p-value <0.05

*Appendix 7-3*: Estimated own- and cross-price elasticities of off- and –on trade beer, cider, wine, spirits and RTDs in the UK for low income population

|  | | Purchase | | | | | | | | | |
| --- | --- | --- | --- | --- | --- | --- | --- | --- | --- | --- | --- |
|  |  | Off-beer | Off-cider | Off-wine | Off-spirits | Off-RTDs | On-beer | On-cider | On-wine | On-spirits | On-RTDs |
| Price | Off-beer | -0.883* | -0.443 | -0.350 | -0.186 | -2.677* | -0.306 | -1.011 | -0.820* | -1.245* | 0.594 |
|  | Off-cider | 0.191 | -1.751* | 0.024 | -0.361 | -0.588 | -0.050 | 0.085 | -0.117 | -0.290 | -0.523* |
|  | Off-wine | -0.094 | 0.483 | -0.472 | -0.111 | 0.449 | 0.037 | -0.851 | 0.436* | -0.778 | 0.210 |
|  | Off-spirits | -0.024 | 0.480 | 0.335 | -0.256 | 0.868 | 0.307* | 1.057* | 0.079 | 0.310 | -0.093 |
|  | Off-RTDs | -0.032 | -0.416* | 0.009 | -0.062 | 0.204 | -0.081 | 0.293 | 0.021 | -0.133 | -0.036 |
|  | On-beer | 0.041 | -0.311 | 0.865* | -0.703 | 1.456 | -0.504 | 3.785* | 0.903 | 1.698* | -3.893* |
|  | On-cider | -0.169 | 0.311 | 0.008 | 0.223 | -0.625 | 0.242 | 0.611 | -0.208 | 0.057 | 0.058 |
|  | On-wine | -0.311 | -0.427 | 0.080 | -0.405 | 0.773 | 0.006 | -1.129 | -0.664* | 1.021 | 0.088 |
|  | On-spirits | 0.111 | 0.295 | 0.029 | -0.152 | 1.176 | 0.166 | 0.063 | 0.324 | -1.108* | 1.126* |
|  | On-RTDs | 0.223 | 0.241 | -0.014 | -0.305 | -0.532 | 0.187 | -0.081 | 0.373* | 0.382 | 0.543 |

Remarks *: p-value <0.05

*Appendix 7-4*: *Estimated own- and cross-price elasticities of off- and –on trade beer, cider, wine, spirits and RTDs in the UK for higher income population*

|  | | Purchase | | | | | | | | | |
| --- | --- | --- | --- | --- | --- | --- | --- | --- | --- | --- | --- |
|  |  | Off-beer | Off-cider | Off-wine | Off-spirits | Off-RTDs | On-beer | On-cider | On-wine | On-spirits | On-RTDs |
| Price | Off-beer | -0.914* | -0.033 | 0.194 | -0.448 | -0.923 | 0.044 | 0.110 | 0.419 | 0.311 | 0.673 |
|  | Off-cider | 0.046 | -1.217* | 0.170 | -0.079 | -0.159 | -0.060 | 0.085 | 0.093 | -0.140 | -0.178 |
|  | Off-wine | -0.017 | 0.775 | -0.417* | 0.359 | -0.138 | -0.331 | -0.020 | -0.097 | -0.085 | 0.019 |
|  | Off-spirits | 0.129 | -0.046 | 0.133 | 0.098 | -0.077 | 0.142 | 0.312 | 0.096 | 0.061 | 0.341 |
|  | Off-RTDs | -0.024 | -0.110 | 0.004 | 0.086 | -0.730* | -0.058 | -0.018 | 0.058 | -0.202 | 0.180 |
|  | On-beer | 0.157 | -0.317 | 0.024 | 0.050 | 0.694 | -0.897* | 0.563 | 0.908* | 1.079* | 0.297 |
|  | On-cider | -0.117 | 0.041 | 0.045 | -0.001 | 0.367 | 0.031 | -0.797* | 0.091 | 0.232 | 0.245 |
|  | On-wine | -0.188 | 0.183 | -0.203 | -0.021 | -0.045 | -0.275 | 0.053 | -0.858* | -0.105 | -0.288 |
|  | On-spirits | 0.025 | -0.212 | -0.036 | -0.347 | -0.386 | -0.039 | -0.499 | 0.057 | -0.783* | 0.898* |
|  | On-RTDs | 0.033 | -0.104 | -0.113 | 0.021 | 0.429 | 0.094 | -0.510 | -0.073 | -0.172 | -0.236 |

*Remarks *: p-value <0.05*
